# Variables Associated with Change in Quality of Life among Persons with Dementia in Nursing Homes: A 10 Months Follow-Up Study

**DOI:** 10.1371/journal.pone.0115248

**Published:** 2014-12-18

**Authors:** Marit Mjørud, Janne Røsvik, Anne Marie Mork Rokstad, Marit Kirkevold, Knut Engedal

**Affiliations:** 1 Norwegian Centre for Ageing and Health, Vestfold Hospital Trust, Tønsberg, Norway; 2 Molde University College, Norwegian Centre for Ageing and Health, Vestfold Hospital Trust, Tønsberg, Norway; 3 Department of nursing science, Institute of health and society, University of Oslo, Oslo, Norway; University Medical Center Rotterdam, Netherlands

## Abstract

**Aim:**

To investigate variables associated with change in quality of life (QOL), measured by QUALID scale and three subscales; tension, sadness and wellbeing, among dementia patients in nursing homes.

**Method:**

A 10 months follow-up study including 198 (female 156, 79%) nursing home patients, mean age 87 (s.d 7.7) years. Scales applied; quality of life in late stage dementia (QUALID) scale and three subscales (wellbeing, sadness and tension), neuropsychiatric inventory questionnaire 10 items (NPI-10-Q), clinical dementia rating (CDR) scale, physical self-maintenance (PSMS) scale and a scale of general medical health. Use of psychotropic medication, gender and age was collected from the patient's records.

**Results:**

Mean baseline QUALID score: 20.6 (s.d.7.0), follow-up score: 22.9 (s.d.7.4), mean change 2.8 (s.d.7.4). QOL improved in 30.8%, were unchanged in 14.7%, deteriorated in 54.6% of patients. A regression analysis revealed that change in QUALID score was significantly associated with: QUALID baseline score (beta -.381, p-value.000), change in NPI score (beta.421, p-value.000), explained variance 38.1%. Change in score on wellbeing subscale associated with: change in PSMS score (beta.185, p-value.019), wellbeing baseline score (beta -.370, p-value.000), change in NPI score (beta.186, p-value.017), explained variance 25.3%. Change in score on tension subscale associated with: change in CDR sum-of-boxes (beta.214, p-value.003), change in NPI score (beta.270, p-value.000), tension baseline score (beta -.423, p-value.000), explained variance 34.6%. Change in score on sadness subscale associated with: change in NPI score (beta.404, p-value.000), sadness baseline score (beta -.438, p-value.000), explained variance 38.8%.

**Conclusion:**

The results imply that a lower baseline score (better QOL) results in a larger change in QOL (towards worse QOL). Change in QOL is mostly associated with change in neuropsychiatric symptoms. In almost 50% of patients QOL did not deteriorate.

## Introduction

Nursing home residents‘ quality of life (QOL) is an important outcome measure in the planning of service provision and as a quality indicator of care in nursing homes (NH) [1], [2]. As persons with dementia (PWD) often reside for several years in nursing homes, it is of importance to know which variables affect changes in quality of life over time [3], [4] in order to effectively maintain or improve these patients' QOL.

Several cross-sectional NH studies using proxy reported QOL have examined the relationship between QOL and neuropsychiatric symptoms (NPS). These studies have shown that NPS, especially depression and anxiety, are associated with reduced QOL [4], [5]. The association between QOL and apathy is unknown [6]–[8]. Proxy reported QOL has also been reported to be associated with cognitive impairment and activity of daily living (ADL) impairment [9]–[12]. However, cross-sectional studies will not necessarily give the same results as longitudinal studies. Therefore, we cannot draw conclusions whether the variables associated with a QOL measure in cross-sectional studies can predict the QOL for PWD in the future to come.

Only a few longitudinal studies have examined how QOL change over time among PWD [3], [13]–[15]. In a two year follow-up study of PWD in a long-term care facility Lyketsos et al. found that QOL decreased, but no association between change in QOL and behavioral disturbance, activities of daily living (ADL), cognition or depressive symptoms was found [3]. The only variable significantly associated with a change in QOL, was the baseline QOL score of the patients. Even thought Lyketsos and coworkers found a small but significant decreases in QOL, about half the participants had unchanged or improved QOL after 2 years [3]. In a 20 month follow-up study of PWD in long-term care facilities, Castro-Monteiro and colleagues found that QOL decreased in more than 50% of the patients. Baseline scores of QOL and number of chronic problems were associated with change in QOL [15]. Selwood and colleagues studied QOL by self-rating in 40 hospitalized persons with dementia in a follow-up study of 13 months. Like Lyketsos and coworkers they found that the only predictor of QOL at follow-up was the baseline QOL. In the Selwood-study almost 45% had unchanged QOL at follow-up and 27% had improved QOL at follow-up [14]. Hoe et al studied PWD residing in care homes in a 20 weeks follow-up study. They reported a decrease in patient-rated QOL in 38.6% of the patients. In this study both patient- and staff- reported QOL were recorded. For the patient-rated QOL they found that a change in QOL was significantly associated with the baseline anxiety and baseline QOL ratings and changes during follow-up in depression, anxiety, cognition, behavior and dependency. Reduced staff-rated QOL was significantly associated with increased dependency, cognitive decline and increased behavioral problems [13].

It is difficult to know whether the place of living makes an impact on QOL. A recent cross-sectional study on QOL in PWD in eight European countries, did not find any differences in QOL between home-dwelling and institutionalized PWD [16]. In this review the presence of depressive symptoms was in most studies associated with lower QOL.

It is difficult to measure the effect of psychotropic drugs on QOL, as psychotropic drugs usually are prescribed because of NPS, assumed to be associated with QOL. Few studies have examined the relationship between use of psychotropic medications and QOL in PWD. One would assume that the use of psychotropic drugs reducing the NPS, would enhance the QOL of PWD. On the other hand, use of psychotropic drugs, especially antipsychotics could have side-effects which can reduce QOL [10], [17].

The four longitudinal studies referred to have used different scales to measure QOL and different sources to obtain information of QOL. None of them have used the quality of life in late stage of dementia scale (QUALID). Further, none have examined the importance of living arrangement in the nursing home. We wanted to use such information in our analyses to prove the robustness of prior studies.

Thus, the aim of this study was to test the following hypothesis: a change in QOL as measured by the QUALID scale and its subscales is associated with the baseline QOL scores, NPS, use of psychotropic drugs, degree of dementia and impairment in activities in daily living, but not with gender and age.

## Method

### Design

This is a 10 months follow-up study of PWD in NH. The data was collected to conduct a cluster randomized controlled trial (RCT) aiming to evaluate the effect of two different methods on how to implement person centered care (PCC) in Norwegian NH [18]. The interventions were implementation of Dementia care mapping in one group of NH and the VIPS practice model in the other group of NH. The patients included in the present study were those randomized to the control group of the RCT, and they received care as usual. In addition, all NH received a DVD with lectures on dementia. In the control group, 10 of 13 units did not use the DVD.

### Participants

We included PWD that were permanent residents in NH, had lived there for at least four weeks and were not terminally ill. All 51 NH in Oslo, Norway with more than 30 beds were invited to participate. Of them 44 units in 16 nursing homes with a total of 899 patients accepted the invitation. One nursing home dropped out before randomization. From this patient sample 721 patients or their next-of-kin consented to participate, and after assessment of dementia 665 patients were included; 56 persons without dementia were excluded. Due to missing data in four cases 661 patients with complete data were included in the analyses of the study. The 661 included patients were randomized into three groups. The patients included in the present study were randomized into the control-group of the study, at baseline a total of 198 patients in five nursing homes. Of them 118 resided in regular nursing home units (RU), whereas 80 lived in special care units (SCU) for PWD.

### Data collection

A standardized interview was used to collect the data. Baseline data was collected in January 2011 and follow-up data in November/December the same year. Research assistants, who were trained in a one day course, collected the data. Most of them had used the instruments of the present study in earlier studies or in daily clinical practice. They collected data from the patients' NH records and interviewed the patients' primary nurses, who were either registered nurses or auxiliary nurses. The project leaders of the study were available during the data collection and could be consulted at any time.

### The variables

The following standardized evaluation scales were applied:

To rate quality of life the Norwegian version of the QUALID scale was used. This is a proxy rated scale used for measuring QOL in persons with moderate and severe dementia. The scale is developed by Weiner and coworkers [19] and consists of 11 items, each tapping an observable type of behavior. Each item is scored between 1 and 5. Thus, the minimum score is 11, which indicates high QOL and the maximum score is 55, which indicates poor QOL. The scale has a good inter-rater reliability and has an acceptable level of internal consistency reliability [19], [20]. It has been validated and tested in Norwegian NH patients and is found to be reliable in this population [9], [21]. According to a principle component analysis the scale has been found to consist of three factors, which can be represented by three subscales; *tension* (the items: physically uncomfortable, verbalization suggests discomfort, irritable and appears calm, minimum score 4, maximum score 20), *sadness* (the items: cries, appears sad and facial expression of discomfort, minimum score 3, maximum score 15) and *wellbeing* (the items: smiles, enjoys eating, enjoys social interaction and enjoys touching/being touched, minimum score 4, maximum score 20) [20], [22].

The clinical dementia rating scale (CDR) was used to rate severity of dementia [23]. The scale consist of six items, and can be used as a categorical variable (0 =  no dementia, 0.5 =  possible dementia, 1 =  mild dementia, 2 =  moderate dementia, 3 =  severe dementia) or as a continuous variable by using the sum of boxes, with scores from 0 =  no dementia, to 18 =  severe dementia. The two scoring systems correlate strongly [24]. The present study used the sum of boxes in the analysis.

The Physical self maintenance scale (PSMS) was used to evaluate the patients' abilities to perform basic activities in daily living [25]. The scale evaluates six different areas (ability to go to the toilet, to eat, to dress, to wash, to walk and to bath), where lower scores indicate better functioning. The scale is a continuous variable with scores ranging from 6 (best) to 30 (worst) [25]. The scale has been used in several large Norwegian NH studies [26]–[28].

The Neuropsychiatric inventory questionnaire with 10 items (NPI-10-Q) was used to assess the severity of behavioral and neuropsychiatric symptoms common in dementia [29], [30]. Each symptom is rated as not present  = 0 or present  = 1. If the symptom is present, the rater will assess severity of the symptom as 1 =  mild, 2 =  moderate and 3 =  severe. Minimum score is 0 and maximum score is 30. I higher score denotes more sever NPS.

The patients general medical health was rated with a four-point global scale, and we used the following ratings: good health  = 1, fair health  = 2, poor health  = 3, very poor health  = 4, taking into account each patient's number of general medical conditions, the severity of those conditions and the use of medication. This instrument is reliable used as a continuous scale [31]. In the study by Lyketsos et al. the ratings were coded the other way around as compared to what we did.

Gender, age, the use of medications and time of stay in the ward were collected from the patient s' records.

### Diagnosis of dementia

Two experienced geriatric psychiatrists independently used all information from the collected data and the patients' NH records to make a dementia diagnoses according to ICD-10. The final diagnoses were made in consensus.

Dementia due to Alzheimer's disease (AD), vascular dementia (VaD), mixed dementia of AD and VaD, dementia due to Parkinson's disease and unspecified dementia (UD) were diagnosed in accordance with the criteria for research of ICD-10 [32]. To diagnose Frontal lobe dementia (FLD) the Manchester-Lund criteria was used [33] and for Lewy body dementia (LBD) the revised consensus criteria was applied [34].

### Ethics statement

Written information was given to the patients and their next-of-kin. The physician and the head nurse of the NH determined whether the patient was competent to give informed consent. When competent, the patient gave written informed consent. For patients lacking ability to give informed consent, the next-of-kin was given the opportunity to decline participation on behalf of the patient based on written information, hence next-of-kin did not provide written consent for participation. The consent procedure was as instructed by the Regional Committee for medical and health research Ethics in South-East Norway. The study and the consent procedure were approved by the Regional Committee for medical and health research Ethics in South-East Norway.

### Statistics

Data were analyzed using the SPSS (statistical program for social science) package, version 19. Distribution of each variable was examined by inspecting histograms, Q-Q and box plots. The Mann-Whitney U-test or the Kruskal-Wallis test was used to test for differences between groups (demographics). Paired sample t-test was used to compare the mean scores on the rating scales between baseline and follow-up.

We checked for inter correlations between the variables, using Spearman's rho. No variables except the QUALID total score and the QUALID subscale scores correlated above 0.5.

After the preliminary analysis showing the distribution of the data and changes in QUALID score between baseline and follow-up (10 month follow-up - baseline), an unadjusted analysis of the associations between changes of QUALID and various patient variables at baseline were performed. Thereafter, we constructed four linear regression analyses using the change in scores (the follow-up score subtracting the baseline score) of the total QUALID score and each of the three QUALID subscales scores as the dependent variables. As independent variables we used the items from the unadjusted analyses that were associated with the four dependent variables with a p-value <0.2. Age, gender and the baseline total QUALID and subscale scores were included in all analyses.

As a second step we conducted another set of four regression analyses using the same dependent variables. As independent variables we used the changes of the scores of the NPI-10-Q, the CDR, the PSMS and use of psychotropic drugs. Age, gender and the baseline total QUALID and subscale scores were also included in these four analyses. We applied both the enter and the backward method in the regression analyses. The results were almost identical and we decided to report the results of the backward method.

As we have data on two levels we considered to use multilevel analysis when performing regression analyses. We therefore checked for cluster effect between the units and calculated the intraclass correlation coefficient (ICC). If the ICC is 5% or higher multilevel analyses should be done to adjust for cluster effect [35]. Otherwise a one-level model can be used. As ICC was only 2.28% we did a one-level model.

## Results

At baseline we included 198 patients with dementia, mean age of 87 (s.d. 7.7) years, 156 (79%) were women. At 10 months follow-up 143 patients still resided in NH. Their mean age was 87.2 (s.d. 7.9) years, 116 (81.7%) were women. Nine persons had moved from NH and 46 were dead. The 46 patients who died before follow-up, differed from the baseline patient group as they were older (p-value 0.036), had more severe dementia (p-value 0.002), worse general medical health (p-value 0.001), being of male gender (p-value 0.002) and more impaired in ADL (p-value 0.010).

At baseline 49 (24.7%) persons had dementia of mild degree as rated by CDR (CDR 1), 73 (37%) had dementia of moderate degree (CDR 2), whereas 76 (38%) had dementia of severe degree (CDR 3). At follow-up 23 (16.1%) persons were in the CDR 1-group, 57 (39.9%) in CDR 2-group and 63 (44.1%) in the CDR 3-group. In the SCU a larger proportion of patients had moderate and severe dementia (85%) compared to those in the RU (68.7%) (chi-square test, p-value 0.022). There were no differences in gender, age, PSMS scores or NPI-10-Q scores between persons living in RU or SCU.

Table 1 presents the mean total QUALID scores for various baseline variables. We found statistically significant differences in QUALID scores between the degrees of dementia, the levels of general physical health, the numbers of neuropsychiatric symptoms, levels of impairment in activities in daily living and whether or not the patients used antipsychotic drugs. Table 2 presents the mean QUALID scores at baseline and follow-up, and in addition the mean changes in scores. Table 2 also presents the proportion of patients that had improved, unchanged or worse score on the total QUALID and the three QUALID subscales. All QUALID scores were significantly higher at follow-up, meaning that qol worsened. Table 3 presents the mean changes in scores between follow-up and baseline regarding the same variables as table 1. For the QUALID total score, only number of NPS and use of anxiolytics were significantly different between follow-up and baseline. For the wellbeing subscale no variables differed between follow-up and baseline. For the sadness subscale, only number of neuropsychiatric symptoms differed between follow-up and baseline. And for the tension subscale, only degree of dementia and use of anxiolytics differed between follow-up and baseline. Table 4 presents changes in scores on NPI-10-Q, PSMS and CDR between baseline and follow-up (follow-up score subtracting the baseline score).

**Table 1:** Baseline characteristics of the 198 patients and QUALID scores of various subgroups of the patients.

		Baseline characteristics	p-value
Gender	Men (n = 42)	20.1 (6.5)	.592^a^
	Women (n = 156)	20.8 (7.1)	
Age, years	<79 (n = 30)	23.2 (6.9)	.137^b^
	80–84 (n = 30)	19.5 (5.4)	
	85–89 (n = 55)	20.1 (7.1)	
	90+ (n = 83)	20.5 (7.3)	
General medical health	Good (n = 31)	18.6 (6.3)	.034^b^
	Fair (n = 101)	19.9 (6.2)	
	Poor (n = 53)	22.5 (8.2)	
	Very poor (n = 13)	23.4 (7.0)	
Type of dementia	Alzheimer's dementia (n = 94)	21.3 (7.4)	.244^a^
	Other dementias (n = 104)	20.0 (6.5)	
CDR score	1 (n = 49)	18.6 (6.3)	.000^b^
	2 (n = 73)	19.0 (6.0)	
	3(n = 76)	23.6 (7.4)	
PSMS score	6–13 (n = 54)	17.9 (5.1)	.000^b^
	14–17 (n = 41)	19.1 (5.9)	
	18–21 (n = 53)	21.6 (7.1)	
	22–30 (n = 50)	23.8 (8.1)	
NPI-Q-10 number of symptoms	0–1 symptoms (n = 69)	16.3 (5.0)	.000^b^
	2–3 symptoms (n = 71)	20.9 (6.2)	
	4–9 symptoms (n = 58)	25.5 (6.8)	
Number of psychotropic drugs on daily basis	0–2 n = 185	20.5 (6.9)	.567^a^
	3–9 n = 13	22.2 (8.6)	
Sedatives	No (n = 165)	20.6 (7.0)	.856^a^
	Yes (n = 33)	20.8 (7.0)	
Antipsychotics	No (n = 169)	20.2 (6.8)	.027^a^
	Yes (n = 29)	23.4 (7.6)	
Anxiolytics	No (n = 165)	20.3 (6.9)	.059^a^
	Yes (n = 33)	22.5 (7.0)	
Antidepressants	No (n = 134)	20.7 (6.8)	.608^a^
	Yes (n = 64)	20.5 (7.5)	
Antidementia	No (n = 182)	20.8 (7.0)	.130^a^
	Yes (n = 16)	18.3 (5.9)	
Type of ward	RU (n = 118)	20.3 (7.4)	.140^a^
	SCU (n = 80)	21.2 (6.3)	
Time in ward	< = 6 months (n = 35)	18.5 (5.7)	.059^a^
	>6 months (n = 163)	21.1 (7.2)	

a Mann-Whitney U-test, ^b^Kruskal-Wallis test, CDR =  Clinical Dementia Rating, PSMS =  Physical Self Maintenance Scale, NPI-Q-10 =  Neuropsychiatric Inventory Questionnaire, 10 items, RU =  regular unit, SCU =  special care unit.

**Table 2:** The total QUALID score and the subscale QUALID scores at baseline and 10 months follow-up for the patients with observations at both time periods (N = 143).

	Baseline scores	10 months scores	Mean change	p-value	Improvement 2 points or more	A change of 0 or 1 point	Worsening 2 points or more
QUALID score (s.d.)	20.6 (7.0)	22.9 (7.4)	2.8 (7.4)	.000	44 (30.8%)	21 (14.7%)	78 (54.6%)
Wellbeing score (s.d.)	7.4 (2.7)	8.5 (3.0)	0.9 (2.9)	.000	31 (21.7%)	48 (33.6%)	64 (44.8%)
Sadness score (s.d.)	5.6 (2.8)	6.2 (3.2)	0.7 (3.3)	.007	28 (19.6%)	62 (43.4%)	53 (37.1%)
Tension score (s.d)	7.2 (3.5)	8.2(3.8)	1.1 (4.0)	.001	28 (19.6%)	51 (35.7%)	64 (44.8%)

**Table 3:** Changes in mean scores, total, wellbeing, sadness and tension QUALID scores, between baseline and follow-up (follow-up – baseline) for various patient characteristics.

		QUALID total score	p-value	Wellbeing score	p-value	Sadness score	p-value	Tension score	p-value
Gender	Men (n = 26)	3.2 (8.0)	.455^a^	0.5 (2.8)	.660^a^	0.6 (2.9)	.922^a^	2.0 (4.6)	.087^a^
	Women (n = 117)	2.7 (7.3)		1.0 (3.0)		0.8 (3.3)		0.9 (3.8)	
Age, years	<79 (n = 24)	2.2 (8.4)	.790^b^	1.5 (3.5)	.665^b^	0.4 (4.0)	.899^b^	0.3 (4.2)	.259^b^
	80–84 (n = 24)	3.7 (7.5)		1.1 (2.5)		0.8 (3.6)		1.8 (4.5)	
	85–89 (n = 38)	2.3 (7.6)		0.9 (2.8)		1.1 (3.2)		0.4 (3.6)	
	90+ (n = 57)	3.1 (6.9)		0.7 (3.0)		0.7 (2.8)		1.7 (3.6)	
General medical health	Good (n = 25)	1.4 (6.7)	.116^b^	0.6 (2.7)	.653^b^	0.2 (3.7)	.239^ b^	0.6 (3.5)	.139^b^
	Fair (n = 81)	3.8 (7.5)		1.2 (2.9)		1.1 (3.0)		1.6 (4.2)	
	Poor (n = 31)	0.7 (7.3)		0.7 (3.0)		−.1 (3.4)		0.1 (3.6)	
	Very poor (n = 6)	5.5 (8.6)		0.5 (3.6)		2.5 (3.7)		2.5 (3.4)	
Type of dementia	Alzheimer's disease (n = 63)	2.5 (8.2)	.831^a^	1.0 (3.2)	.948a	0.8 (3.9)	.770^a^	0.7 (3.8)	.251^a^
	Other dementia (n = 80)	3.1 (6.8)		0.9 (2.7)		0.7 (2.7)		1.5 (4.1)	
CDR score	1 (n = 40)	3.6 (6.4)	.493^b^	0.7 (2.9)	.845^b^	0.7 (2.4)	.810^ b^	2.2 (3.9)	.033^b^
	2 (n = 57)	3.3 (7.6)		1.2 (2.8)		0.6 (3.5)		1.4 (3.8)	
	3 (n = 46)	1.6 (8.0)		0.8 (3.1)		0.9 (3.7)		−.2 (3.9)	
PSMS score	6–13 (n = 41)	2.5 (6.1)	.566^b^	0.7 (2.4)	.603^b^	0.5 (3.1)	.921^ b^	1.3 (3.7)	.581^b^
	14–17 (n = 33)	4.4 (7.8)		1.8 (3.3)		0.7 (3.0)		1.9 (3.6)	
	18–21 (n = 42)	2.4 (7.8)		0.9 (2.9)		0.9 (3.2)		0.6 (4.5)	
	22–30 (n = 27)	2.1 (8.3)		0.4 (3.2)		0.9 (3.9)		0.7 (3.9)	
NPI-Q-10 number of symptoms	0–1 symptoms (n = 56)	4.9 (6.4)	.042^b^	1.3 (3.1)	.384^b^	1.7 (2.5)	.009^b^	1.6 (3.2)	.376^b^
	2–3 symptoms (n = 48)	1.9 (7.7)		0.5 (2.7)		0.6 (3.3)		0.8 (4.5)	
	4–9 symptoms (n = 39)	1.1 (7.8)		0.9 (2.9)		−.4 (3.7)		0.5 (4.1)	
Number of psychotropic drugs on daily basis	0–2 (n = 136)	2.7 (7.4)	.543^a^	0.9 (2.9)	.992^a^	0.7 (3.3)	.415^a^	1.1 (4.0)	.295^a^
	3–9 (n = 7)	4.5 (7.1)		0.7 (3.0)		2.3 (3.3)		2.6 (2.9)	
Sedatives	No (n = 122)	2.7 (7.6)	.607^a^	0.9 (3.0)	.947^a^	0.7 (3.2)	.720^a^	1.1 (4.1)	.538^a^
	Yes (n = 21)	3.7 (6.4)		1.0 (2.4)		1.1 (3.6)		1.6 (3.2)	
Antipsychotics	No (n = 117)	2.9 (7.5)	.894^a^	0.9 (3.0)	.943^a^	0.7 (3.3)	.842^a^	1.2 (3.8)	.996^a^
	Yes (n = 26)	2.6 (7.3)		0.9 (2.9)		0.8 (3.2)		0.8 (4.5)	
Anxiolytics	No (n = 119)	3.5 (7.6)	.022^a^	1.0 (3.0)	.508^a^	1.0 (3.3)	.060^a^	1.5 (4.0)	.019^a^
	Yes (n = 24)	−.33 (5.8)		0.6 (2.5)		−.3 (3.1)		−.5 (3.6)	
Antidepressants	No (n = 93)	2.5 (6.8)	.349^a^	0.8 (2.8)	.667^a^	0.8 (3.2)	.918^a^	1.0 (3.8)	.346^a^
	Yes (n = 50)	3.4 (8.5)		1.2 (3.1)		0.7 (3.4)		1.4 (4.3)	
Antidementia	No (n = 131)	2.8 (7.5)	.991^a^	1.0 (3.0)	.152^a^	0.7 (3.2)	.358^a^	1.1 (4.0)	.869^a^
	Yes (n = 12)	2.9 (6.3)		0.0 (1.7)		1.6 (3.6)		1.3 (3.2)	
Type of ward	RU (n = 88)	2.6 (7.4)	.484^a^	0.8 (3.0)	.423^a^	0.5 (3.0)	.189^a^	1.3 (4.1)	.876^a^
	SCU (n = 55)	3.1 (7.4)		1.1 (2.7)		1.1 (3.7)		0.9 (3.8)	
Time in ward	< = 6 months (n = 24)	3.4 (6.9)	.957^a^	0.6 (2.3)	.532^a^	1.3 (3.4)	.671^a^	1.6 (3.3)	.837^a^
	>6 months (n = 119)	2.7 (7.5)		1.0 (3.0)		0.6 (3.2)		1.1 (4.1)	

a Mann-Whitney U-test, ^b^Kruskal-Wallis test, (N = 143).

**Table 4:** Change in scores on Neuropsychiatric Inventory-10 Questionnaire (NPI-Q -10), the Physical Self Maintenance Scale (PSMS) and the Clinical Dementia Rating scale, sum of boxes (CDR), between baseline and 10 months follow-up (N = 143).

	Baseline score	10 months score	Mean change	p-value
NPI-Q-10 score, mean (s.d.)	4.6 (4.6)	6.1 (5.0)	1.4 (5.3)	.002
PSMS score, mean (s.d.)	17.1 (4.9)	19.2 (4.2)	2.1 (3.6)	.000
CDR sum, mean (s.d.)	12.2 (3.7)	13.3 (3.7)	1.1 (3.4)	.000

The results of the first four multiple linear regression analyses resulted in few significant associations and low explained variance.

For change in total QUALID score we included baseline total QUALID score, age, gender, general physical health, number of NPS and use of anxiolytics. Only the variable “baseline total QUALID score” was significantly associated with the change in QUALID score (beta −.447, p-value.000, explained variance 19.4%). This means a lower baseline score gave a larger change.

For change in the tension subscale score we included baseline tension score, age, gender, general somatic health, use of anxiolytics and CDR score. The tension baseline score (beta −.454, p-value.000) and CDR score (beta −.159, p-value.033) were significantly associated with the change of the tension score (explained variance 24.5%) This means a lower baseline tension score and a lower baseline CDR gave a larger change at 10 months.

For change in the wellbeing subscale score we included baseline wellbeing score, age, gender and anti-dementia medication. The baseline wellbeing score was the only significant variable (beta −.445, p-value 0.000, explained variance 19.4%) This means a lower baseline score gave a larger change.

For change in the sadness subscale score we included baseline sadness score, age and gender, type of ward, number of NPS and use of anxiolytics. The baseline sadness score (beta −.486, p-value.000) and type of ward (beta −.125, p-value 0.029) were of significance (explained variance 22.7%). Again, a lower baseline score gave a larger change, and PWD living in SCU had more deterioration of QOL as compared to the PWD in RU.

The second set of multiple regression analyses using changes in the scores of the NPI-10-Q, CDR and PSMS in addition to the baseline scores of the total QUALID and the baseline subscale scores, use of psychotropic drugs, age and gender as independent variables, are presented in table 5. Using the changes in scores of the variables gave a larger explained variance for each of the QUALID scores (both total and subscales).

**Table 5:** Variables associated with a change in QUALID scores (total and subscale scores).

		Unadjusted analyses		Adjusted analysis	
		Beta	p-value	Beta	p-value
**Change in QUALID total score**	QUALID baseline score	−.397	.000	−.381	.000
	Change in NPI-Q-10 score	.532	.000	.421	.000
	Anxiolytics at baseline	−.192	.021	−.082	.249
	Change in PSMS score	.170	.042	.101	.161
	Adjusted R square	38.1%			
**Change in QUALID Wellbeing**	Change in PSMS score	.232	.005	.185	.019
	Wellbeing baseline score	−.370	.000	−.370	.000
	Change in NPI-Q-10 score	.354	.000	.186	.017
	Adjusted R square	25.3%			
**Change in QUALID Sadness**	Change in NPI-Q-10 score	.469	.000	.404	.000
	Sadness baseline score	−.379	.000	−.438	.000
	Adjusted R square	38.8%			
**Change in QUALID Tension**	Change in CDR score	.239	.004	.214	.003
	Change in NPI-Q-10 score	.391	.000	.270	.000
	Tension baseline score	−.358	.000	−.423	.000
	Anxiolytics at baseline	−.196	.019	−.026	.733
	Change in PSMS score	.173	.023	−.008	.924
	Adjusted R square	34.6%			

Linear regression analyses (backward method).

## Discussion

Our hypothesis was partly confirmed. Both the first and second set of regression analyses found that changes in QOL after 10 months as measured by the QUALID scale and its subscales were associated with the baseline scores for the total QUALID and the three subscales (see text in the results section and table 5). A lower score at baseline (better QOL) was associated with worsening at 10 months. Age, gender and general somatic health were not associated with a change in QOL.

The results of both sets of linear regression analysis (referred to in the text and table 5), shows that the baseline total QUALID score and the subscale scores are all negatively associated with the changes in scores between baseline and 10 months follow-up. This means that a lower baseline score (indicating better QOL), results in a larger change of QOL (indicating a worsening of QOL) after 10 months. This is not unexpected and is in line with results from other studies that have reported a reduction in QOL over time [3], [13], [15], [36]. Hoe and colleagues and Vogel and colleagues also found that a well preserved quality of life at baseline, would predict reduction in QOL over time [13], [36]. However, we observe in table 2 that almost half of the patients (45.5%) had unchanged or improved QOL after 10 months. This is also similar to the results of prior longitudinal studies [3], [14].

The second set of regression analyses (table 5) investigating variables associated with change in QOL revealed that change in severity of NPI-10-Q score was significantly associated with change in QOL. The baseline score of the NPI-Q-10 was on the other hand not associated with a change in QOL (first set of analyses). This indicates that an increase in the severity of neuropsychiatric symptoms will lead to decreased QOL, but also that we cannot predict the future QOL by considering the NPI score at baseline. The result is not unexpected, as both longitudinal studies [13], [17] and cross-sectional studies have found neuropsychiatric symptoms to be associated with reduced QOL [7], [10], [12], [37]. Worsening of NPS can be caused by the progression of dementia [38]. However, the progression of dementia can hardly be the only explanation for the association between worsening of QOL and worsening of NPS over time, as the CDR score did not influence the change of the total QUALID score during follow-up. NPS, maybe especially agitation, might also be understood as communication, and hence, we should try to understand what it is PWD are trying to communicate, to increase wellbeing and reduce depression and agitation [18].

As seen in table 5, the independent variables associated with change in the sadness subscale, change in NPI score (beta .404) and the sadness baseline score (beta −.438) explained 38.8% of the variance. Again, a lower baseline score gave a larger change. We have argued elsewhere that the sadness subscale holds signs of depression (crying, appearing sad and facial expression of discomfort) [12]. As shown in table 2, the sadness score was significantly worse after 10 months. This was a bit surprising, as we by face validity of its items expected that the QUALID sadness subscale score should be a measure of depression. Other studies have found depression to be stable [39], [40] or to decrease [38] through the course of dementia. On the other hand, 63% of the PWD had no change or improved sadness score at 10 months. This indicates that a smaller group of PWD, in this case 37% of all, had a larger worsening in the QUALID sadness score. In the first set of regression analysis, the change in the sadness subscale was associated with type of ward. Living in a SCU in a NH was associated with a larger change in the sadness subscale. This was a bit unexpected, because the SCUs are specially designed for PWD in order to improve QOL of the patients. A possible explanation of the association may be that PWD living in SCU in the present study were more cognitively impaired, than those living in RU. The study by Abrahamson and colleagues also found mood-scores to be lower in SCU than in regular units [41], [42].

A change in the tension subscale score was associated with both the baseline CDR score (reported in the text) and the changed score of CDR (table 5), and as already reported, the baseline tension score. Level of cognitive impairment is found to be associated with QOL in other studies using proxy-based information [13], [36]. However, the results from other studies are for the total scores of QOL, and not for subscale scores like the tension subscale.

In the unadjusted analysis we found that impairment in ADL was associated with the change in the total QUALID score, the tension score and the wellbeing score. But except for the change in the wellbeing score, this association disappears in the adjusted analysis. As seen in table 5, change in the wellbeing score was associated with a change in ADL (beta .185). A lower baseline wellbeing score (indicating better QOL) and increased dependency in ADL gave a larger change, meaning worse QOL. This is similar to the results of Barca and colleagues' cross-sectional study, which found that impairment in ADL was associated with the “comfort subscale” [9]. Hoe and colleagues also found increased dependency to be associated with change in QOL [13]. However, their result is not especially related to wellbeing.

As can be seen in table 1, presenting baseline results of all 198 patients there is a significant difference in the total QUALID score between the persons using and those not using antipsychotics. This difference disappears when we analyzed the same difference among those who resided in NH after 10 months (table 3), indicating that the PWD having worse QOL and using antipsychotics were among those who died during follow-up. However, we found an association between the use of anxiolytics and a change in total QUALID and tension subscale scores, and an almost significant association for the change in the sadness scores. In the unadjusted analysis reported in table 5, the change in the total QUALID score and the tension subscale score were associated with use of anxiolytics. In an earlier cross-sectional study we also found that anxiolytics and antipsychotics were associated with these two scores [12]. However, after controlling for the changes in NPI-Q-10, CDR and ADL these associations vanished in the adjusted analysis. Ven-Vakhteeva and colleagues studied the impact of use of psychotropic drugs, antipsychotics in particular, on QOL [17]. Our results are in line with the results of that study. After adjusting the analyses for a change of NPS the use of psychotropic drugs was not significantly associated with QOL [17]. On the other hand Ven-Vakhteeva and coworkers found an improvement of QOL in the persons who discontinued the use of anxiolytics [17]. As findings from various studies are contradictive we will suggest that the relationship between the use of psychotropic drugs and QOL in PWD is still uncertain, and should be studied further, because such a relationship could have important clinical implications.

Neither gender nor age was associated with change in QOL. These results are in line with prior longitudinal studies [3], [17]. The review on QOL by Beerens, also suggests there are no association between socio-demographics and QOL [4].

General somatic health was not associated with QOL, as measured by QUALID. A few studies have shown QOL to be related to general physical health [43], [44]. Our negative results regarding such an association could indicate that the QUALID scale is an inadequate tool to measure QOL in physically ill elderly people, and is a dementia specific scale.

The explained variance in the first set of analyses was about 20%. By including the change in scores on the various assessment scales the explained variance increased, indicating that the associations shown in table 5 between the independent variables and the outcome variables have a stronger explanatory power than the regression analyses described in the text. Still, for the wellbeing subscale the explained variance only increased to 25%, indicating that wellbeing is not well explained by the variables included in this study. A review by Bjørkløf and colleagues on coping and depression, concludes that coping style and resources of the person are associated with fewer symptoms of depression [45]. Wellbeing might also be influenced by coping and life expectation in the PWD, and frailty in the PWD might direct the staffs attention towards physical care, instead of focusing on how to increase happiness and wellbeing in PWD through for instance individualized activities [46].

The baseline QUALID scores had a strong influence on the change in QOL in the regression analysis. This might be a result of regression to the mean even though we controlled for other variables.

As many of the PWD included in this study have unchanged or even improved QOL at 10 months (45.5%), this may reflect on the subjective nature of QOL. Many people adapt to situations of living with chronic diseases, and this might even be possible in situations like moving into and living in NH. The subjective nature of QOL, the use of proxy data and of different evaluation scales might cause the different results that we see in various studies. As there is no agreement on what QOL is, how to best measure QOL in PWD and what dimensions should be included in a measurement [2], [47], [48], it is difficult to find the exact nature of and variables associated to QOL.

## Limitations and Strengths

A limitation of the study is related to the fact that our data is based on proxy reports. Hence, we do not know if it truly reflects the QOL of PWD. The staff evaluation of QOL in PWD might be biased by their own expectations in life and their view on dementia. Also, the staff might have been biased by watching the DVD they received. Another limitation is the relatively small number of participants. A strength of the study is the use of validated scales, the longitudinal design, and to the best of our knowledge, the first study examining changes in the QUALID subscales. We claim these results add new information about QOL of PWD residing in NH.

## Conclusion

The results of the study implies that a lower baseline score (indicating better QOL) as measured with the QUALID scale results in a larger change towards deterioration in QOL over time. Change in QOL is mostly associated with the change in NPS. However, in almost 50% of the patients QUALID score did not deteriorate.
